# The costs associated with stroke care continuum: a systematic review

**DOI:** 10.1186/s13561-023-00439-6

**Published:** 2023-05-17

**Authors:** Jorgina Lucas-Noll, José L. Clua-Espuny, Mar Lleixà-Fortuño, Ester Gavaldà-Espelta, Lluïsa Queralt-Tomas, Anna Panisello-Tafalla, Misericòrdia Carles-Lavila

**Affiliations:** 1 Department of Primary Care, Institut Català de La Salut, Av. de Cristòfol Colom, 20, Tortosa, Tarragona 43500 Spain; 2 University Institute for Primary Health Care Research Jordi Gol I Gurina (IDIAPJGol), Barcelona, Spain; 3 Department of Nursing, Universitat Rovira I Virgili, Tarragona, Tarragona Spain; 4 Department of Economic and Bussiness, Universitat Rovira I Virgili, Tarragona, Tarragona Spain

**Keywords:** Acute stroke, Costs study, Healthcare, Pre-hospital pathways, No-treatment cost

## Abstract

Stroke, a leading cause of death and long-term disability, has a considerable social and economic impact. It is imperative to investigate stroke-related costs. The main goal was to conduct a systematic literature review on the described costs associated with stroke care *continuum* to better understand the evolution of the economic burden and logistic challenges. This research used a systematic review method. We performed a search in PubMed/MEDLINE, ClinicalTrial.gov, Cochrane Reviews, and Google Scholar confined to publications from January 2012 to December 2021. Prices were adjusted using consumer price indices of the countries in the studies in the years the costs were incurred to 2021 Euros using the World Bank and purchasing power parity exchange rate in 2020 from the Organization for Economic Co-operation and Development with the XE Currency Data API. The inclusion criteria were all types of publications, including prospective cost studies, retrospective cost studies, database analyses, mathematical models, surveys, and cost-of-illness (COI) studies. Were excluded studies that (a) were not about stroke, (b) were editorials and commentaries, (c) were irrelevant after screening the title and abstract,(d) grey literature and non-academic studies, (e) reported cost indicators outside the scope of the review, (f) economic evaluations (i.e., cost-effectiveness or cost–benefit analyses); and (g) studies not meeting the population inclusion criteria. There may be risk of bias because the effects are dependent on the persons delivering the intervention. The results were synthetized by PRISMA method. A total of 724 potential abstracts were identified of which 25 articles were pulled for further investigation. The articles were classified into the following categories: 1)stroke primary prevention, 2) expenditures related to acute stroke care, 3) expenditures for post-acute strokes, and 4) global average stroke cost. The measured expenditures varied considerably among these studies with a global average cost from €610-€220,822.45. Given the great variability in the costs in different studies, we can conclude that we need to define a common system for assessing the costs of strokes. Possible limitations are related to clinical choices exposed to decision rules that trigger decisions alerts within stroke events in a clinical setting. This flowchart is based on the guidelines for acute ischemic stroke treatment but may not be applicable to all institutions.

**Supplementary Information:**

The online version contains supplementary material available at 10.1186/s13561-023-00439-6.

## Introduction

Stroke is a leading cause of death and long-term disability and have a considerable social and economic impact as the second highest disease burden in Europe [1]. A 34% increase of stroke is predicted in the coming decades, the number of ischemic strokes recorded in people above 80 years of age will triple (2010–2060), and an estimated 27% increase of stroke survivors will also increase expenditure on health and non-health care of stroke, which was 1.7% of overall health expenditure [2–5].

Most of the literature on stroke-related costs has focused on short-term [6], in-hospital expenditures [5] with a relative scarcity of studies [7] focused on long-term stroke-related components such as urban vs. non-urban territory, long-term ambulatory care, medical expenses, informal care, caregiver burden, events related to poor risk factors control, and social care services. Also, several authors have calculated projections of stroke for specific regions, countries [8–12], or internationally [13, 14], combining demographic projections with estimated future incidence and mortality rates or obtaining them by extrapolation from past trends [8–10, 12–14]. Therefore, there are great differences in the methods used for calculating costs, conditioned by the issues included as preventive costs of cardiovascular factors, especially hypertension (HTA) and atrial fibrillation (AF) [1, 15], the cost of new treatments such as thrombolysis and thrombectomy in early critical care (Code Stroke) [16, 17], and dependency management costs [18] after the episode; there are also differences in the calculated cost criteria [19–21] (direct vs. indirect) and in the final format of the result (total cost, average cost per stroke, average cost per person vs. mortality reduction benefits). Guidelines from different organizations are mostly similar and when differences exist, they are typically in the strength of the recommendation made about newly published data, with some guideline committees endorsing new protocols, whereas others require a higher level of data before making a strong recommendation [22]. Consequently, there are significant differences among countries [15] regarding treatment accessibility and costs, and improvements and effective strategies would have a massive beneficial impact on healthcare systems [23]. Also the complexities of finding the right balance between data privacy and access to data for health services research makes it difficult to compare the obtained data and the convergence in the calculation methods.

It is an imperative to investigate those new issues associated to stroke care economic burden and logistic challenges according the goal outlined by the Stroke Action Plan for Europe for the management of acute stroke that “every person with acute stroke deserves the right to have equal access to optimized and efficient stroke care, diagnosis and treatment, regardless of the place of living, age, gender, culture, social and economic status” [15].

The aim of this research is to conduct a systematic literature review on the described costs associated with stroke care *continuum* to better understand the evolution of the economic burden and logistic challenges.

## Methods

The method used in this systematic review consisted of a search strategy, inclusion and exclusion criteria, data extraction, and quality assessments of included studies. The study was performed according to the Preferred Reporting Items for Systematic reviews and Meta-Analysis (PRISMA) guideline [16].

### Search strategy

A comprehensive literature search was performed for full-text papers in the PubMed/MEDLINE, ClinicalTrial.gov, Cochrane Reviews [24], EconLit, and Ovid/EMBASE databases confined to publications in the last 10 years (January 1, 2012–December 31, 2021). We augmented the search by using Google Scholar and checking the references of the articles we obtained (Additional file 1: Appendix Table 1). Additional articles were added from systematic reviews using the snowball citation method. The systematic search was carried out in January–February 2022. The terms were matched with terms in the Medical Subject Heading(MeSH) database and included the possible combinations of stroke, atrial fibrillation, hypertension, cost analysis, economics, hospital charges, reimbursement, fees, cost-of-illness, social care charges, indirect costs, economic burden, informal caregiving, and dependency cost as [stroke and cost analysis] or [stroke and economics] or [stroke and hospital charges] or [stroke and social care charges] or [stroke and reimbursement] or [stroke and fees] or [stroke and cost-of-illness] or [stroke and indirect cost] or [stroke and economic burden] or [stroke and informal caregiving] or [stroke and dependency costs] or [cost and atrial fibrillation] or [stroke cost and atrial fibrillation] or [stroke cost and hypertension] or [stroke cost and blood pressure control] or [stroke cost and atrial fibrillation and hypertension].

### Analysis and sources

We limited the search to titles and abstracts in a first approximation of human studies, published in English, and clinical trials or reviews. Subsequently, for the selected studies, search strategy development, searching, screening, application of criteria, selection, appraisal, data extraction, and synthesis were completed by at least two investigators. Disagreements were resolved by consensus. For study design and publication type, the inclusion criteria were all types of publications, including prospective cost studies, retrospective cost studies, database analyses, mathematical models, surveys, and cost-of-illness (COI) studies. The initial review of titles and abstracts excluded studies that (a) were not about stroke, (b) were editorials and commentaries, (c) were irrelevant after screening the title and abstract,(d) grey literature and non-academic studies, (e) reported cost indicators outside the scope of the review, (f) economic evaluations (i.e., cost-effectiveness or cost–benefit analyses); and (g) studies not meeting the population inclusion criteria.

According to previous evidence and the ESO Guideline Directory (mobile stroke units, acute stroke management, secondary prevention and rehabilitation, and long-term consequences) [25], four categories of costs were investigated: (1) effective preventive treatment to reduce the likelihood of people suffering strokes, such as risk factors associated with their incidence, especially AF and HTA, by primary care; (2) early critical care (Stroke Code) to minimize stroke damage and reduce the likelihood of disability by multi-disciplinary stroke units; (3) post-stroke care and after hospital discharge including secondary cardiovascular prevention and socio-health care at home or in a long-stay institution that estimates the value of all resources spent or foregone, including health care cost and productivity loss, due to stroke and the cost of caregiving by the replacement (RA) approach, also known as the proxy good method. Global cost-of-illness (COI) studies included both direct and indirect cost analyses [26]. De-duplication strategy was applied by EndNote and Mendeley technics.

#### Analysis of direct cost

Costs related to the health care system: preventive care (AF and HTA), outpatient care, emergency care, hospital care, rehabilitation care, patient transportation inside the health system (ambulance, medical units), and pharmacological prescriptions. Standard medical care for strokes included early assessment, time-critical therapy, and stroke unit care. A cost for care provided by formal health care providers such as a home health aide is considered a direct cost.

Costs related to social care: the cost of residual disability after a stroke presents a major economic and humanistic burden and is not considered part of the care given by formal health care providers.

Informal care costs as well as non-medical costs outside the health system such as transfer of patients, informal care, or other expenses borne by the patient. The cost of informal caregiving is the value of the time spent by family members or other caregivers that is not considered part of the care given by formal health care providers. Non-medical costs outside the health system such as transport and time of patients and caregivers, assistive devices, or other expenses borne by the patient.

#### Analysis of indirect costs

Productivity costs: result from loss of paid and unpaid work and replacements due to premature death (mortality cost) and the cost of disability due to the reduced productivity of stroke survivors (morbidity cost).

The data analysis was carried out by collecting and synthesizing information on general study characteristics, methodology (study design, data sources, approaches, and calculated disease burden indicators), and estimated economic burdens (currency and year, cost components, cost perspective). Information collection was carried out by descriptively focusing on the burden of disease due to stroke at the household, health system, and community levels. Costs of healthcare will increase with forecasted inflation rates, so the current value of cost estimates should be adjusted. To compare costs of different countries in different study periods, we derived the current value in the article by using consumer price indices of the study countries in the years of the study and in 2021 from the World Bank and purchasing power parity (PPP) exchange rate in 2020 from the Organization for Economic Co-operation and Development (OECD) with the XE Currency Data API [27].

The Scottish Intercollegiate Guidelines Network (SIGN) grading system [28] checklist for systematic reviews was used to rank economic studies. The methodology behind the system is based on a set of variables that recognize key factors, especially bias and confounding variables, that can influence the quality of a study or its conclusion [29].

## Results

**Fig. 1:**
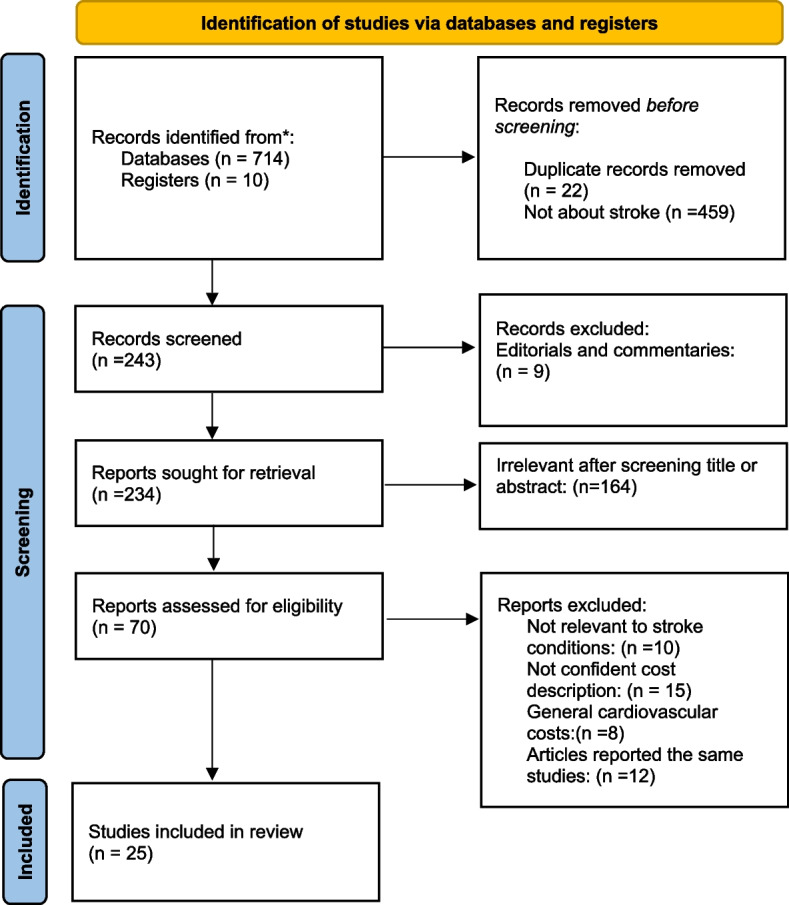
Flow-chart of studies through review process

Figure 1 shows the Preferred Reporting for Systematic Reviews and Meta-Analysis (PRISMA) flowchart that summarizes the selected studies. A total of 724 potential abstracts were identified; 369 from MEDLINE via PubMed, 131 from Cochrane, and 224 from Google Academics. Of the 724 abstracts, 481 were excluded because they weren’t about strokes, 9 were excluded because they were editorials and commentaries, and 211 were excluded because they were obviously irrelevant after screening the title and abstract. We classified the 25 publications pulled for further investigation into four groups.

A summary of the studies according to stroke-related costs are described in Table 1.

**Table 1:** Characteristics of included studies according to evaluated cost

	**HEALTH CARE SYSTEM COST**				**COST Nonrelated with Health CARE SYSTEM Cost**		**PRODUCTIVITY COSTS**	
**Author, year, country**	**PP**		**ACUTE STROKE**	**POST ICTUS**	**SOCIAL CARE**	**PATIENTS AND CAREGIVERS COST**	**MORBIDITY LOSSES**	**MORTALITY LOSSES**
	**HTA**	**AF**						
Wodchis WP, 2011 (Canada) [30]		X						
Cotte,2016 (France) [31]		X						
López-López 2017, (UK) [32]		X						
Giner-Soriano, 2020 (Spain) [33]		X						
Zhang, 2017, (EEUU)[34]	X							
Gheorghe, 2018 (UK) [35]	X							
Kostova, 2020 (EEUU) [36]	X							
Salvatore, 2021 (Italy) [29]	X	X						
Shireman TI,2017 (EEUU) [37]			X					
Zhang H, 2019 (China), [38]			X	X				
Abdo RR, 2018 (Leban)[20]			X					
Reimer AP, 2019 [39]			X					
De Andres 2014 (Spain) [40]			X	X				
Van Meijeren-Pont W, 2016 [41]			X	X				
Oliva-Moreno, 2013 (Spain) [42]						X		
Skolarus, 2016 (EEUU) [43]						X		
Joo H, 2017 (EEUU) [3]						X		
Alvarez-Sabin, 2013, (Spain) [44]			X	X			X	
Van Eeden, 2015 (Netherlands) [45]				X	X	X	X	X
Xu. 2018 (UK) [46],			X	X	X	X	X	X
Benjamin. 2019 (EEUU)[4]			X	X			X	X
Luengo-Fernandez, 2020 (UK) [2],			X	X	X	X	X	X
Patel A, 2020(UK) [47]			X	X	X	X	X	X
Rochmah TN., 2021 (Indonesia) [48]			X	X	X	X	X	X
Stefan Strilciuc, 2021 Romania [49]			X	X	X	X	X	X

^*^PP Primary Prevention, *HTA* Arterial Hypertension, *AF* Atrial Fibrillation

### Stroke primary prevention (Table 2)

**Table 2:** Expenditures related to stroke primary prevention

First Author Year of publication Country	Objectives	Methods				Results
		**Design and** **GRADE LEVEL **[32]	**Participants**	**Instruments**	**Calculated stroke costs**	
Wodchis WP, 2011 (Canada) [30]	Review and synthesize the literature on the costs of atrial fibrillation (AF)	Systematic review **1 + GRADE**	115 articles	FA related direct costs	Direct Costs	Average cost: 4750 € (1224€—15,824 €)
Cotte, 2016 (France) [31]	To investigate the annual burden of cardiovascular complications in AF	Retrospective longitudinal population-based study **2 + GRADE**	533,044 AF patients	Stroke related to AF hospitalizations, global burden	Direct Costs	Cost per patient: Hemorrhagic stroke (€12,748), ischemic stroke (€11,234), Systemic Embolism (€9087), unspecified stroke (€8108), and Transient Ischemic Attack (€3734)
López-López, 2017 (UK), [32]	To compare Direct Oral Anti-Coagulants (DOACs) with each other and with warfarin for prevention of stroke in patients with AF	Systematic review, network meta-analysis, and cost effectiveness analysis **1 + GRADE**	94,656 patients with AF 23 randomized trials involving	Cost effectiveness of preferred licensed products	Direct Costs	Total average cost per patient/year was between €23,064 from €24,841 per patient /year
Giner-Soriano, 2020 (Spain), [33]	To analyze the use, effectiveness, safety and costs of stroke prevention in AF in patients with dabigatran or vitamin K antagonists (AVK)	Observational study of population-based cohorts **2 + GRADE**	14,930 patients with AF	Consumption of healthcare resources and costs associated with the management of atrial fibrillation	Direct Costs	Average cost of €4075.60 in the group treated with dabigatran Average cost of €4551.40 in the AVK group
Zhang, 2017 (EEUU) [34]	To summarize evidence on cost effectiveness of community-based interventions to control hypertension based on a review	Systematic review **1 + GRADE**	64 articles	-cost-effectiveness analysis; -cost–benefit analysis	Direct Costs	Average cost €51,5 ( €33–€94,05) for 1-mmHg reduction in systolic blood pressure (SBP) and €11,538 ( €5513-€48,353) for 1 life-year gained Screening interventions cost from €17,930 to €46,818 in the U.S., €505 to €4650in Australia, and €5755 to €14,850 in China
Gheorghe, 2018 (UK), [35]	To Synthetize the economic burden of Cardiovascular Disease (CVD) and hypertension	Systematic review **1 + GRADE**	83 articles	Total costs for HTA treatment in each CVD	Direct Costs	Average costs for hypertension:€18,15 per month
Kostova, 2020 (EEUU) [36]	Review and synthesize the literature on the costs of HTA	Systematic review **1 + GRADE**	70 articles	HTA related direct costs	Direct Costs	The range of the annual intervention per hypertension patient was from €5,16 for a non-drug program to €2014,2 for a Pharm program
Salvatore, 2021 (Italy)[29]	To investigate the costs of cardiovascular disease in the regional health service in Italy's from 2014 to 2016	Retrospective longitudinal population-based study **2 + GRADE**	98,829 patients	Cardiovascular complications related to AF hospitalizations, global burden	Direct Costs	Atrial Fibrillation costs range from €3724,67 to €4328,88

Ninety percent of strokes are related to modifiable risk factors. Among them pointed out the AF and HTA [1, 8, 32] all both largely undiagnosed and uncontrolled. Most of the studies estimated treatment costs, but not the costs of its approach and control due to stroke events. The annual treatment of a patient with AF has an average cost between €4,750 and €23,064/patient/year [33]. About one-third of these costs could be attributed to anticoagulation management [30]. If cardiovascular complications are included [31], the average cost may increase to around €11,234/patient/year.

On the other hand, it is known that there is a direct relationship between HTA and risk of stroke and that its control reduces the risk of stroke [29]. According to the studies in the review, the annual cost per patient with HTA is highly variable depending on the origin of the population studied and the therapy administrated [33, 34]. The range of the costs goes from €5 in patients who only receive dietary advice to more than €2,000/patient/year if they have pharmacological treatment [3, 36].

### Acute care for a stroke (Table 3)

**Table 3:** Articles about cost related to acute care stroke

First Author Year of publication Country	Objectives	Methods				Results
		**Design** **GRADE LEVEL **[32]	**Participants**	**Instruments**	**Calculated stroke costs**	
Shireman TI, 2017 (EEUU) [37]	Demonstrate improved 90-day outcomes for patients with acute ischemic stroke treated with stent retriever thrombectomy plus tissue-type plasminogen activator (SST + tPA) compared with tPA	Prospective economic study **2 + GRADE**	196 patients	Cost-effectiveness from the perspective of the US healthcare system	Direct costs	Total 90-day costs: - SST + tPA: €52,468,5 - tPA: €41,171
Abdo RR, 2018 (Lebanon) [20]	To investigate the direct Medical Cost of Hospitalization	Prospective Incidence-Based Multicenter **2 + GRADE**	230 patients	Cost-of-Illness Study	Direct costs	The average in-hospital cost per stroke patient was €5798,15 ± €113,047
Zhang H, 2019 (China), [49]	To investigate the total direct hospitalization costs of stroke. These costs include laboratory and diagnostic costs, medications costs, bed fees and services	Cross-sectional study from 2006 to 2013 **2 + GRADE**	114,872 hospitalizations for five stroke types	Databases of Guangzhou City for the years 2006 through 2013	Direct costs	The average cost of hospitalization for stroke patients was €2328,77
Reimer AP, 2019 (EEUU)[39]	To perform a cost consequence analysis of standard transport (ST) vs. Mobile Stroke Unit (MSU)	Cost-Consequence Analysis of Mobile Stroke Units vs. Standard Prehospital Care and Transport	Standard emergency medical services between July 2014 and October 2015 for 400 patient transports MSU	Cleveland Clinic Mobile Stroke Unit	Direct costs	The incremental cost of the MSU vs. ST was €86,134 cost of air transfer (> €11,303.30)
De Andres, 2014 (Spain) [40]	To estimate the use of healthcare resources and costs of acute Cardioembolic Stroke management	Estudio CODICE. multicenter prospective observational study **2 + GRADE**	128 patients	Spanish National Health System	Direct costs	Average total cost per patient during acute-phase management and rehabilitation was €13,139
Van Meijeren-Pont W, 2016 (Netherlands) [41]	To estimate societal costs up to one year after the start of rehabilitation	Stroke Cohort Out-comes of REhabilitation (SCORE) study	313 stroke patients	2014 to 2016	Mean cost out-patient rehabilitation for 12 months	Mean costs for inpatient and outpatient rehabilitation were €86,133 and €33,517 respectively

**Fig. 2:**
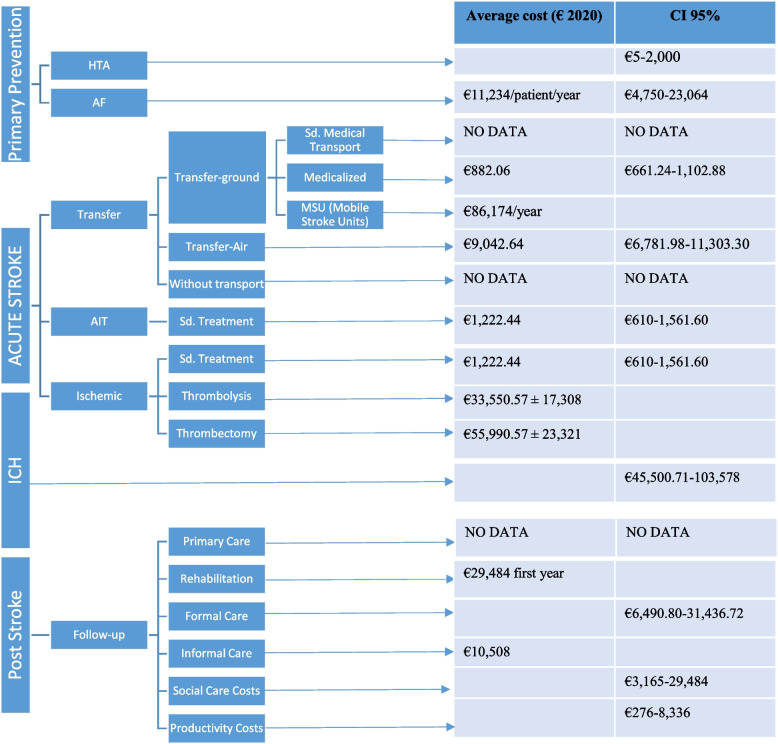
Global stroke cost from the results of the studies included in the current review. The figure illustrates clinical choices exposed to decision rules that trigger decisions alerts within stroke events in a clinical setting. This flowchart is based on the guidelines for acute ischemic stroke treatment but may not be applicable to all institutions

The average healthcare cost of a stroke per person, including early assessment, time-critical therapy, and stroke unit care, rehabilitation, and follow-up care, is estimated as an average of €5,798.15–€140,048 [20, 35, 48]. Regarding stroke types, Intracerebral Hemorrhage (ICH) was the most expensive, followed by Ischemic Stroke (IS), and Transient Ischemic Attack (TIA) [48, 49]. The Fig. 2 shows the flow-chart of the attending process from what have been calculated the cost issues.

#### Transfer to stroke care unit vs emergency department

It is of special interest due to the characteristics of non-urban and rural regions vs door-to-needle times. There are different ways: standard transfer to the closest local stroke *center* (in geographical areas where the default health authority assigned referral stroke center is a non-thrombectomy capable hospital where the individual may be treated with thrombolysis or standard medical care)vs Direct Transfer to Endovascular Center (a direct transfer of selected candidates to an endovascular center bypassing the nearest local hospital).This fact challenges the geographic equity in the access to thrombectomy. In both scenarios, time to treatment initiation is critical and the sooner the treatment is started, the higher are the chances of clinical recovery. The transfer could be made by standard medical transport or medicalized transfer-ground [cost €882.06 (661.24–1102.88), mobile stroke units (MSUs) [cost additional €86,174/year], and medicalized transfer-air [cost €9,042(6,781.58–11,303.30)] [38, 39, 50] according to local emergency medical services transport protocols.

*Standard medical care* in stroke includes early assessment, CT scan [€308.66 (231.8–385.52], time-critical therapy and emergency department visit [€913.78 (686.86–1,176.08)] [39]

*Fibrinolysis [rt-PA(Activase)]*used for improving neurologic recovery and reducing the incidence of disability in adults with acute IS. The average cost of rt-PA was €33,550.57 ± 17,308/patient [51]

*Mechanical thrombectomy (MT)*: several studies have demonstrated the superiority of thrombolysis plus MT vs. thrombolysis alone to treat strokes [37, 52, 53]. The evidence demonstrates that performing MT up to 24 h after acute IS symptom onset is still cost-effective, suggesting that this intervention should be implemented based on improving quality of life as well as economic grounds resulting in a dominant therapy over rt-PA alone. Average cost of MT was €55,990.57 ± 23,321. Thrombectomy was associated with an increase in initial hospital cost of €22,440/patient compared with rt-PA alone [51].

The acute costs of stroke were variable according to the type of stroke suffered, as well as the treatment performed. The costs are different if the patient receives standard or conservative treatment, or an interventional treatment. In the case of* intracerebral hemorrhage, by the severity and comorbidities frequently associates, have a* highest burden by treatment, rehabilitation and support services. The average costs range from €45,500.71–73,367.55/patient [48]. Many organizations, such as the European Stroke Organization and The Stroke Alliance for Europe, have prioritized primary prevention for this disease [54].

The mean total cost per patient for the management of acute stroke was €13,138.97. The hospital stays (€5,916.62) and rehabilitation (€3,835.40) were the resources that contributed the most to this total cost [55].

### Post-acute stroke (Table 4)

**Table 4:** Articles about cost related to care post-stroke

First Author Year of publication Country	Objectives	Methods				Results
		**Design and** **GRADE LEVEL **[32]	**Participants**	**Instruments**	**Calculated stroke costs**	
Oliva-Moreno, 2013 (Spain) [42]	Estimate the social cost of non-professional (informal) care provided to survivors of cerebrovascular accidents (CVA)	Prospective economic study **2 + GRADE**	329,544 people	2008 Survey on Disability, Independent Living and Dependency (EDAD-08)	Indirect costs calculating each hour of care at 12.71 euros	The estimated hours of informal care provided in 2008 amounted to 852 million €
Skolarus, 2016 (EEUU) [43]	To investigate the annual cost of stroke caregiving	Cross sectional study **2 + GRADE**	892 patients	National Health and Aging Trends Study (NHATS), a nationally representative survey of Medicare beneficiaries	Indirect costs calculating each hour of care at 6,55 euros/hour	Average annual cost for caregiving for an elderly stroke survivor was €8712 22.3 h/week
Joo H, 2017 (EEUU) [3]	To describe the Economic burden of informal caregiving associated with stroke	Systematic Review **1 + GRADE**	10,129 patients 6 articles	2010 Health and Retirement Study, data on non-institutionalized adults aged ≥ 65 years	Indirect costs calculating each hour of care at 10,54 euros/hour	€4332 per year (95% CI: €1685-€6514) for informal caregiving

The two main factors that determine spending in the first year are hospitalizations and convalescence/rehabilitation. Hospitalizations accounted for 45% of the difference in spending between the first year after the stroke and the year before, while convalescence/rehabilitation accounted for 33% [56].

Rehabilitation is very important to reduce mortality. It used to be done by a multidisciplinary team [16]. Patients with greater disability (Rankin 3–5: 41.8%) have a higher cost than patients without disability (Rankin 0–2: 20.8%), or €8,500 vs. €6,000 [55]. There is large variability in access to rehabilitation depending on the different sanitary systems. The first-year post-stroke costs are €29,484 on average. For the third year and onward, follow-up cost equal to cost of the second year, but rehabilitation remains a major part of the cost [40, 41]. Europe-wide implementation of home-based rehabilitation was estimated to be cost-effective (> 90%) compared to center-based rehabilitation [57] but the domiciliary service was found to cost 2.6 times more.

#### Secondary cardiovascular prevention

23% of patients who has suffered a stroke will suffer a second stroke with more disability (36% to 51%) and increased mortality (20% to 34%). Secondary prevention measures have the potential to reduce the number of stroke survivors having additional strokes by 80% [2]. Most factors are modifiable, and stroke risk prediction with machine learning techniques (MLT) has a significant ability to predict the risk of stroke occurrence, so it highlights the potential value of expanding the use of MLT in clinical practice. Nowadays, only 40% of patients with HTA after a stroke were correctly treated, and there are many patients with AF that don’t receive Oral anticoagulant Therapy (OAT). In the review period, there is no information about the cost of the prevention of a new stroke and/or the cardiovascular factors related to.

#### Socio-sanitary (formal care)

Up to 50% of survivors were chronically disabled [18, 45]. Most stroke survivors return from the hospital to community or residential care settings where many receive help with activities of daily living (ADLs) and instrumental activities of daily living (IADLs). The range of costs varied from €6,490.80 to €31,436.72/patient/year [58] statistically significant differences in the cost of formal care were found according to patients’ degrees of dependency and depending on whether all elderly stroke survivors were considered or only those with stroke-related health problems.

#### Non-healthcare system costs

##### Social care costs

Non-medical costs outside the healthcare system such as accompaniment to physician appointments, transportation, assistive devices, or other expenses borne by the patient. Residual disability after a stroke presents a major economic and humanistic burden. The interval cost is between €3,165–29,484/patient/year [42, 43, 58].

##### Patients’ and caregivers’ informal care costs

An estimated average of time-care provided by friends or family (who would have provided the same type of caregiving services)with an average cost €10,508/patient/year [42, 43, 58].

##### Productivity costs

Include the loss of paid and unpaid work and replacements due to premature death (mortality cost) and the cost of disability because of the reduced productivity of stroke survivors (morbidity cost) associated with the replacement of workers including productivity losses due to the substitution of workers or the training costs of new employees. This has a range of cost between €276 and €8,336/patient/year [2].

### Global average stroke cost (Table 5)

**Table 5:** Articles about global cost related to stroke

First Author Year of publication Country	Objectives	Methods				Results
		**Design and** **GRADE LEVEL**[32]	**Participants**	**Instruments**	**Calculated stroke costs**	
Alvarez-Sabin, 2013 (Spain) [44]	To find the cost of stroke in Spain	Epidemiological, observational, prospective, multicenter study **2 + GRADE**	321 patients	Sociodemographic, clinical, and economic data	Direct costs Indirect costs	Total average cost per patient/year was €27,711. Direct healthcare costs €849 patient/year and non-healthcare costs to an average of €18,643patient/year. Productivity loss costs per patient/year were €276
Van Eeden, 2015 (Netherlands) [45]	Explore over 1 year poststroke the societal costs, changes in costs and quality of life (QoL) and the relation between costs and QoL	Epidemiological, observational, prospective, multicenter study **2 + GRADE**	395 patients	Cost-of-illness (in Euros) and QoL (in utilities) of a disease	Direct costs Indirect costs	Indirect cost = €11,416 Direct cost = €18,068 Total societal costs for 1 year poststroke were €29 484
Xu, 2018 (UK), [46]	To estimate patient-level health and social care costs of stroke for all patients admitted to hospital with stroke in UK	Epidemiological, observational Retrospective Health economic simulation **2 + GRADE**	111,846 patients	Health Care Costs Productivity Costs	Direct costs Indirect costs	The estimated stroke cost varied widely according to patient characteristics, ranging from €8149 to €49,922 at one year and €21,259 to €119,464 at five years
Benjamin. 2019 (EEUU) [4]	To estimate the cost of Cardio Vascular Diseases and risk factors	Statistical update **2 + GRADE**	18,462 patients	Health interview surveys	Direct costs Indirect costs	Direct care €6673 /patient/year Post ictus Care €9412/patient/year
Luengo-Fernandez. 2020 (UK) [2]	To provide an estimate of the overall economic costs of stroke for all 28 countries of the EU	Population-based cost analysis **2 + GRADE**	1,5 million patients 32 European countries	Population-based cost analysis	Direct costs Indirect costs	Health Care: €17,728 /patient/year Social Care: €3165/patient/year Productivity costs: €8336/ patient/year Informal care costs: €10,508/ patient/year Total costs: €39,736,66 /patient/year
Patel A, 2020 (UK) [47]	To estimate 2014/2015 annual cost from a societal perspective	Epidemiological retrospective study **2 + GRADE**	331 patients	Annual mean cost per person and aggregate UK cost of stroke for individuals aged ≥ 40 Health and social costs	Direct costs Indirect costs	Mean cost of new-onset stroke is €61,665 (95% CI €57,109-€66,220) in the first year after stroke and €33,648 (€27,477-€39,819) in subsequent years
Rochmah TN, 2021 Indonesia [48]	To analyze the burden of stroke: analyzing the average length of hospitalization, and the magnitude of economic losses due to stroke	Systematic review **1 + GRADE**	13 articles	Cost of illness studies that assess burden of stroke in primary and referral healthcare (hospital-based) Direct and indirect costs	Direct costs Indirect costs	The economic burden of stroke disease based on cost of illness method, which is approximately equal to € 1478- €265,613 (direct costs 86.2%, and indirect costs 13.8%)
Stefan Strilciuc, 2021 Romania[49]	To compile the results of existing studies on the economic burden of stroke,	Systematic review **1 + GRADE**	forty-six articles published in twenty-three countries between 1994 and 2019	Across all cost perspectives	Overall costs	Costs in the per patient per year category varied from €103,578 in South Korea (for hemorrhagic stroke) to €610 in Singapore

Table 5 presents a summary of studies reporting global average costs of stroke in the long term (more than 30 days post-stroke). Many these studies were retrospective analyses of a sample of Medicare patients hospitalized for ischemic stroke. The measured expenditures varied considerably among these studies with an average cost from €610-€220,822.45/patient. Seventy-four percent of the costs in the first year post-stroke was in the first six months [41]. Average per patient per year costs is greater in high-income countries.

Costs vary considerably between patients in the first five years post-stroke. They are highest in patients with ICH, older patients, and those with more severe strokes. Over time the costs of providing social care account for a greater proportion of the total care costs of stroke. Prevention transfer pre-hospital services, thrombolysis, thrombectomy and early supported discharge services are cost-effective and cost-saving, with economic savings to healthcare and social care costs of stroke [3, 46].

## Discussion

Most cost-analysis studies conducted thus far have focused on short-term, in-hospital costs of a stroke. Most of these studies are retrospective analyses of different inpatient care databases, and due to diversity in reporting, a detailed cost analysis addressing different segments of services was not possible. Therefore, there is a need for in-depth research using large databases [47]. Stroke incidence is set to increase by 32% in 2035 and 41% in 2040 in addition to long-term disability among stroke survivors [42]. Updated treatment guidelines and improved detection of strokes remain the cornerstone [59].

### Stroke primary prevention

Only primary care performance in stroke care is associated with lower hospital costs [60] but despite the progress in AF diagnosis and management, prevention of strokes remains the cornerstone. The clinical benefits of appropriate anticoagulation are widely recognized, and clinicians should be aware of the importance of anticoagulation therapies in stroke prophylaxis, the occurrence of stroke, and the downstream economic burden on an increasingly aging population [61]. AF quintuples the risk of stroke. Ischemic stroke occurring in patients with AF ≥ 75 year-old was 2.3 times higher and one-fourth ≥ 80 year-old will suffer an AF [62]. The 23.5% with known AF were not receiving OAT. The AF was associated with more severity, disability and 20% increase in stroke-related costs [63].

Recently [64] Cardiology Associations have proposed adding the costs, both in healthcare resources and in social burden, due to stroke events associated with poor control of anticoagulants in AF, which would increase the mean costs significantly. It must be added to overall cost of stroke, €295/year/patient with atrial fibrillation and not-treating and/or inappropriate anticoagulation treatment.

Regarding HTA, the estimation in the overall population of the costs per mmHg reduction does not seem to be a comparable methodology given the difficulty of having reference values and, especially, the variability in blood pressure measurements, treated or not. Therefore, the cost estimate provided by the different studies is neither homogeneous nor does it include all the possible factors and, therefore, its results would be difficult to generalize. Each risk factor would modify the complexity in cost estimation and the future seems to be associated with the development of machine learning techniques to assess the effect severity and unmeasured confounding.

### Acute stroke care

The long-term severity and progression of cerebral infarction before and after recanalization is modified by triage strategy and prehospital pathways, and these have not been evaluated enough for complexity and accessibility. The cost can vary significantly by clinical [65] and geographic factors. However, there were no significant cost differences between direct admission and secondary transfer (€214.55- 781.30) [66]. Among the publications that focused on direct costs, there was a greater emphasis on emergency and short-term care, and less on preventive care (AF and HTA as factors related to stroke incidence), outpatient, rehabilitation, and nursing home care. The greatest determinants of costs in year one was hospitalization, representing 48.5% of the overall costs [66].

Given that the published data confirmed that thrombectomy-treated patients were significantly more likely to be functionally independent than those receiving standard care, early critical care has become a keystone of acute ischemic stroke therapy, but also accounted for nearly one-third of the total expenditure [58, 67–71]. The costs of selection for Endovascular thrombectomy (EVT) have rarely been studied [69, 70] beyond interventions representing costs made in consecutive years post-stroke. Every 10 min of earlier treatment by pre-hospital triage and in-hospital workflow to endovascular thrombectomy may be €11,968.93 (€6,9589.47–18,027.94) of net monetary benefits for taking health care and societal perspectives over a disability-free life [72] . Consequently, health care policies to implement efficient pre-hospital triage and accelerate in-hospital work-flow are needed.

### Post-stroke care

Of the patients who have a stroke, 26.3% will need a caregiver, but the costs are higher in the first year due to the expense of hospital admission and rehabilitation, which represent 33% of the costs. After year one, the increase in expenditure seems mainly determined by additional hospital admissions and drug treatment, [17, 71] but new data [64] showed that the increase in the economic burden (health and social costs) is mainly associated with social care (300%) after one year post-stroke (€10,204.9–€31,964.9/patient/year) and up to five years post-stroke vs. a mean health cost increase (€15,728.9–€20,994.2/patient/year).

Comprehensive rehabilitation and personalized care and support for as long as the person may need should be possible. However, it has been shown that [58] the number of hours of help received by stroke survivors is higher than in prior studies. Under base case assumptions, home-based rehabilitation was found highly likely to be cost-effective (> 90%) compared to center-based rehabilitation in most European countries [57].

### Global average stroke cost

The total costs varied significantly as a result of the heterogeneous cost variables described [68]. In Europe the most common cause of death is morbidity and associated disability [73]. The main forms of CVD are ischemic heart disease (IHD) and stroke [74]. Stroke mortality has been declining since the early twentieth century.

The total costs of strokes in 2017 in Europe were €60 billion [2]; 62% of the costs were for inpatient hospital care, 18% for outpatient care, 13% for primary care, 5% for pharmaceuticals, 3% for emergency care, and 8% more were spent on social care systems. As of now, approximately 34% of the global total healthcare expenditure is spent on stroke [75]. These costs are especially high in older patients, in patients with greater severity, and those with hemorrhagic stroke. Patients with early thrombolysis or early rehabilitation reduced their health and social care costs by five years after stroke. Figure 2 shows the global stroke cost obtained from the results of the studies included in the current review.

Few studies have considered the indirect costs of stroke, including productivity loss due to morbidity and mortality, and costs of patients and caregivers usually provided by unpaid family members, although the indirect costs have been claimed to be large. It has been found that productivity cost estimates vary remarkably according to the choice of method in many disabling diseases such as stroke, and the results of monetary amounts are disease-specific, as seen when comparing the productivity costs in different stroke subtypes. The methodology for estimating lost productivity is an area of considerable debate. Existing literature regarding the economic burden of stroke is concentrated in high-income settings. There are necessary adjustments, especially related to vacancy durations and macroeconomic conditions, and we encourage future studies to make use of routine data to generate more accurate productivity estimates for strokes.

In the Stroke Action Plan for Europe a goal was outlined for the management of acute stroke that “every person with acute stroke deserves the right to have equal access to optimized and efficient stroke care, diagnosis and treatment, regardless of the place of living, age, gender, culture, social and economic status” [16], but the inclusion of adjustment costs may produce a more stringent cost-effectiveness analysis criterion, may affect the relative priority of interventions, and make consensus impossible even on ordinal rankings of health states [65]. Further studies are required to quantify the cost-effectiveness or cost savings of these interventions independently and when combined in regional strategies and community networks of stroke care.

## Conclusions

1. There is a wide interval in the information on the global costs of strokes with an average between €610-€220,822.45 based on the cost of the illness.
2. There is high variability in the relative percentage of costs related to each evolutionary stage of stroke: primary prevention (8.8%), acute stroke (31.67%), and post-stroke (49.4%).
3. The greatest determinant of cost was inpatient hospital care, representing 44.49% of overall costs, but the increase in the economic burden (health and social care costs) is mainly associated with social care (300%) after one year post-stroke.

Given the great variability in costs in the different studies, it would be convenient to define a common schedule for assessing the stroke costs to obtain comparable results. This variability may be related to differences among cost factors included, the monetary value, the offered services included in each health system, and the complexity of access to data from health services.

## Supplementary Information

**Additional file 1. Appendix Table 1.** Search strategy, terms and descriptors used. **Appendix Table 2.** Scottish Intercollegiate Guidelines Network (SIGN) grades of evidence. **Appendix Figure 1.** Stroke chart-flow and cost stroke organization.

## Data Availability

Not applicable.

## Abbreviations

HTA: Hypertension
AF: Atrial Fibrillation
MeSH: Medical Subject Heading
COI: Cost-of-illness
RA: Replacement Approach
PPP: Purchasing Power Parity
OECD: Organization for Economic Co-operation and Development
SIGN: Scottish Intercollegiate Guidelines Network
PRISMA: Preferred Reporting for Systematic Reviews and Meta-Analysis
PP: Primary Prevention
DOACs: Direct Oral Anti-Coagulants
AVK: Vitamin K Antagonists
CDV: Cardiovascular Disease
ICH: Intracerebral Hemorrhage
IS: Ischemic Stroke
TIA: Transient Ischemic Attack
SST: Stent Retriever Thrombectomy
tPA: Tissue-type Plasminogen Activator
ST: Standard Transport
MSU: Mobile Stroke Unit
SCORE: Stroke Cohort Out-comes of Rehabilitation
MT: Mechanical Thrombectomy
CVA: Cerebrovascular Accident
NHATS: National Health and Aging Trends Study
MLT: Machine Learning Techniques
OAT: Oral Anticoagulant Therapy
ADLs: Activities of Daily Living
QoL: Quality of Life
EVT: Endovascular Thrombectomy
IHD: Ischemic Heart Disease
