# Supplementary material for: The costs associated with stroke care continuum: a systematic review
Source: Health Econ Rev. 2023 May 17;13:32. doi: 10.1186/s13561-023-00439-6 (PMC10190015; doi:10.1186/s13561-023-00439-6)
Supplement: Supplementary file 1 — Additional file 1. Appendix Table 1. Search strategy, terms and descriptors used. Appendix Table 2. Scottish Intercollegiate Guidelines Network (SIGN) grades of evidence. Appendix Figure 1. Stroke chart-flow and cost stroke organization. [file 13561_2023_439_MOESM1_ESM.docx]

**Appendix.**

***Table 1: Search strategy, terms and descriptors used***


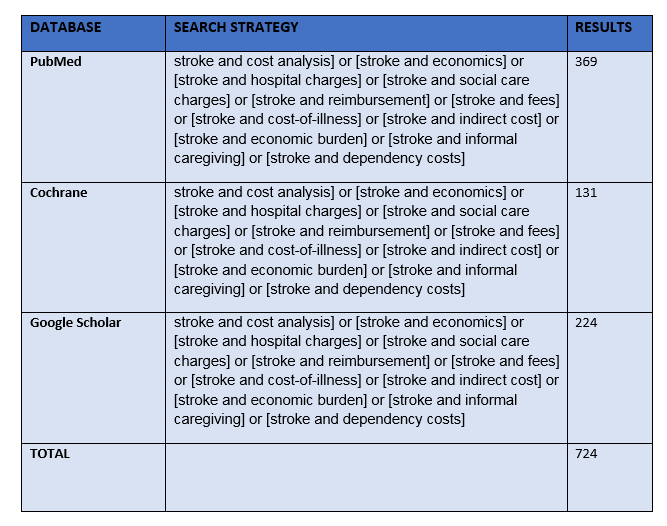


***Table 2: Scottish Intercollegiate Guidelines Network (SIGN) grades of evidence.***
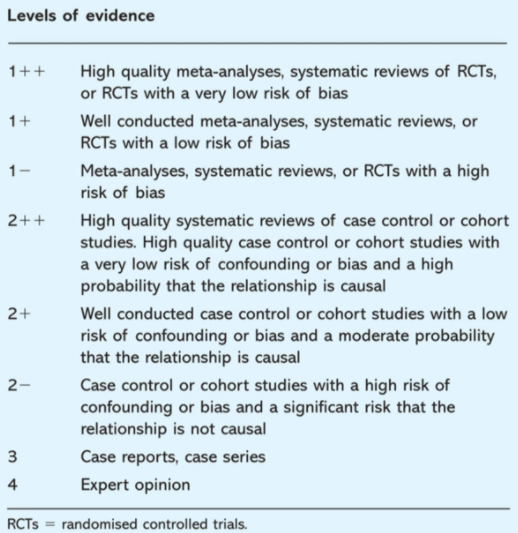


***Figure 1: Stroke chart-flow and cost stroke organization***
